# Cold-Inducible RNA Binding Protein as a Vaccination Platform to Enhance Immunotherapeutic Responses against Hepatocellular Carcinoma

**DOI:** 10.3390/cancers12113397

**Published:** 2020-11-16

**Authors:** Leyre Silva, Josune Egea, Lorea Villanueva, Marta Ruiz, Diana Llopiz, David Repáraz, Belén Aparicio, Aritz Lasarte-Cia, Juan José Lasarte, Marina Ruiz de Galarreta, Amaia Lujambio, Bruno Sangro, Pablo Sarobe

**Affiliations:** 1 Centro de Investigación Médica Aplicada (CIMA), Universidad de Navarra, 31008 Pamplona, Spain; lmsilva@unav.es (L.S.); jegea@unav.es (J.E.); lvlegarda@alumni.unav.es (L.V.); mruiz@unav.es (M.R.); diallo@unav.es (D.L.); dreparaz@alumni.unav.es (D.R.); baparicio.1@alumni.unav.es (B.A.); alasarte.2@alumni.unav.es (A.L.-C.); jjlasarte@unav.es (J.J.L.); 2 IdiSNA, Instituto de Investigación Sanitaria de Navarra, 31008 Pamplona, Spain; bsangro@unav.es; 3 Centro de Investigación Biomédica en Red Enfermedades Hepáticas y Digestivas CIBEREHD, 31008 Pamplona, Spain; 4 Department of Oncological Sciences, Icahn School of Medicine at Mount Sinai, New York, NY 10029, USA; marina.ruizdegalarreta@mssm.edu (M.R.d.G.); Amaia.Lujambio@mssm.edu (A.L.); 5 Liver Unit, Clínica Universidad de Navarra, 31008 Pamplona, Spain

**Keywords:** cold-inducible RNA binding protein, therapeutic vaccination, hepatocellular carcinoma, immune checkpoint inhibitors

## Abstract

**Simple Summary:**

Current immunotherapies based on blockade of immunosuppressive elements provide limited results in liver cancer patients. Here we tested whether combination of this therapy with a vaccine based on the Cold-Inducible RNA Binding Protein (CIRP) would improve its efficacy. Combination of immunotherapy with a CIRP-based vaccine increased vaccine immunogenicity and, when tested in several mouse models of liver cancer, resulted in better therapeutic effects. Despite good immune responses observed in peripheral organs, lymphocytes infiltrating the tumor appeared exhausted, with a weak functional capacity. Finally, by using the same strategy, we prepared a new CIRP-based vaccine containing glypican-3, human antigen commonly found in patients with liver cancer. An equivalent combination enclosing this new vaccine was also highly immunogenic. This suggests that CIRP-based vaccines may enhance the beneficial effects provided by current immunotherapies. However, they should also consider incorporating new elements to overcome limitations observed in tumor lymphocytes.

**Abstract:**

Therapies based on immune checkpoint inhibitors (ICPI) have yielded promising albeit limited results in patients with hepatocellular carcinoma (HCC). Vaccines have been proposed as combination partners to enhance response rates to ICPI. Thus, we analyzed the combined effect of a vaccine based on the TLR4 ligand cold-inducible RNA binding protein (CIRP) plus ICPI. Mice were immunized with vaccines containing ovalbumin linked to CIRP (OVA-CIRP), with or without ICPI, and antigen-specific responses and therapeutic efficacy were tested in subcutaneous and orthotopic mouse models of liver cancer. OVA-CIRP elicited polyepitopic T-cell responses, which were further enhanced when combined with ICPI (anti-PD-1 and anti-CTLA-4). Combination of OVA-CIRP with ICPI enhanced ICPI-induced therapeutic responses when tested in subcutaneous and intrahepatic B16-OVA tumors, as well as in the orthotopic PM299L HCC model. This effect was associated with higher OVA-specific T-cell responses in the periphery, although many tumor-infiltrating lymphocytes still displayed an exhausted phenotype. Finally, a new vaccine containing human glypican-3 linked to CIRP (GPC3-CIRP) induced clear responses in humanized HLA-A2.01 transgenic mice, which increased upon combination with ICPI. Therefore, CIRP-based vaccines may generate anti-tumor immunity to enhance ICPI efficacy in HCC, although blockade of additional checkpoint molecules and immunosuppressive targets should be also considered.

## 1. Introduction

Hepatocellular carcinoma (HCC), the dominant form of primary liver cancer, is a leading cause of cancer and of cancer death worldwide [1]. Although different etiologies have been described as responsible for generating this tumor (chronic infections caused by hepatitis B and C viruses, alcohol abuse, obesity, etc) all of them have in common the presence of chronic liver inflammation [2]. However, despite this role of the inflammatory response in promoting tumor development, the same immune response may exert a beneficial antitumor effect. Indeed, higher antitumor T-cell responses have been reported in patients at earlier stages, as opposed to individuals with advanced disease, and the presence of T-cells has been associated with longer survival [3]. Moreover, T-cell infiltration correlates with reduced tumor recurrence following resection and liver transplantation [4,5]. These data suggest that strategies promoting the generation/enhancement of antitumor immunity may have a therapeutic effect. In this sense, immunotherapies based on the administration of immune checkpoint inhibitors (ICPI), antibodies blocking molecules that downregulate lymphocyte functions, have demonstrated impressive effects in cancer patients, including those with HCC. Thus, initial studies using antibodies targeting CTLA-4 [6] or PD-1 [7,8] were carried out in HCC patients, leading to the FDA-approval of anti-PD-1 antibodies. More recently, combinations targeting PD-L1 and VEGF [9], CTLA-4 and PD-1 [10] or PD-1 plus the multikinase inhibitor lenvatinib [11] have shown superior response rates. However, as in other tumors, there is still a large proportion of patients with HCC who do not respond to these therapies, indicating that additional strategies have to be implemented to increase the response rate. Among other factors, the lack of tumor-specific lymphocytes has been proposed as an important cause of failure for checkpoint inhibitors [12]. Indeed, we have recently reported that a trend for higher responses to the PD-1 inhibitor Nivolumab in HCC patients is associated with the presence of infiltrating T-cells and with inflammatory gene signatures [13]. This suggests that activation of antitumor immunity may overcome this problem and combination of ICPI with protocols generating an immune response would enhance the efficacy of already approved immunotherapies.

In this context of immune enhancement, vaccines have been traditionally considered as a specific strategy to promote antitumor immune responses [14]. Nevertheless, despite their capacity to activate immunity, their clinical efficacy has been limited, presumably due to the immunosuppressive tumor environment [15]. ICPI not only target molecules expressed by tumor-infiltrating lymphocytes, but they may also contribute to promoting immunogenicity of vaccines by inhibiting mechanisms operating during initial T-cell priming [16,17]. Therefore, our aim was to develop a vaccine to be combined with ICPI, enhancing thus the response rate against HCC obtained with these drugs.

We recently reported a new vaccination platform based on the inflammatory factor cold-inducible RNA-binding protein (CIRP) [18]. CIRP is an endogenous TLR4 ligand released under stress conditions that induces the production of inflammatory cytokines [19]. When conjugated to peptide antigens, CIRP-containing immunogens induce CD8-specific responses able to reject established tumors. Therefore, we hypothesized that conjugation of relevant liver tumor antigens to CIRP may generate a vaccine amenable to combination with ICPI for HCC treatment. Here we demonstrate that combination of a CIRP-based vaccine with ICPI not only increases vaccine potency but also, when tested in a therapeutic preclinical setting, leads to higher antitumor effect than ICPI alone.

## 2. Results

### 2.1. Immunogenicity of a CIRP-Based Vaccine Harboring a Polyepitopic Antigen Is Enhanced by Combination with ICPI

**Figure 1:**
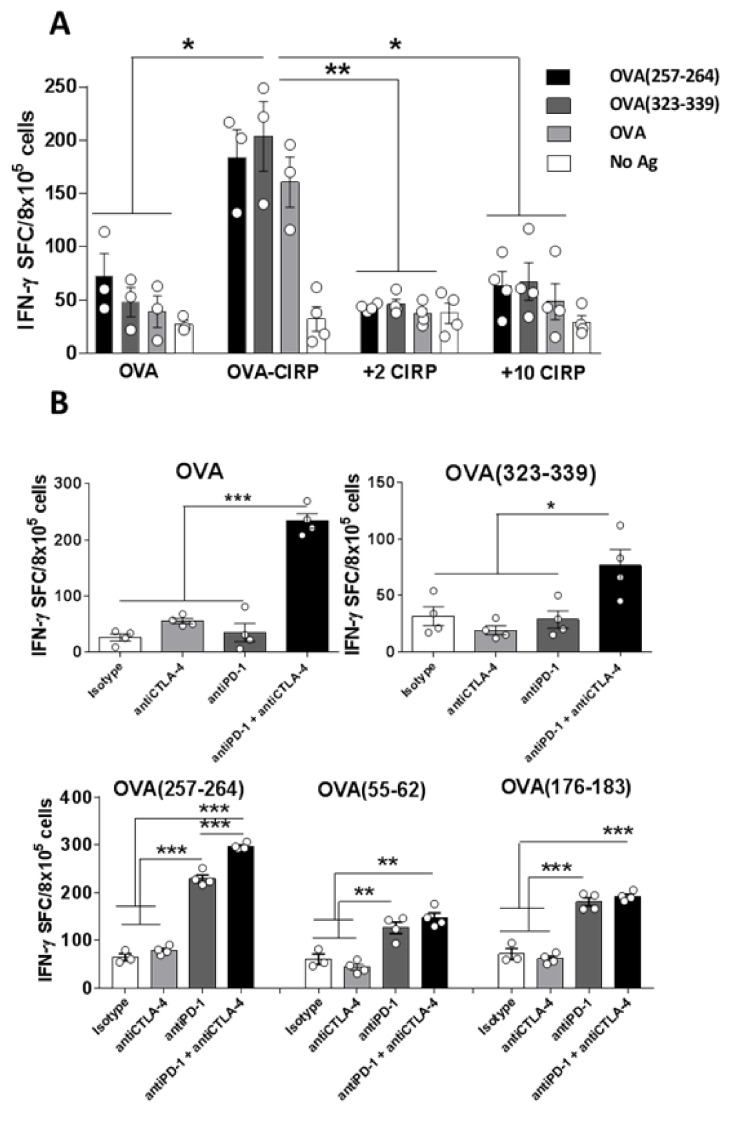
Conjugation of ovalbumin (OVA) to the CIRP platform induces polyepitopic T-cell responses that are enhanced by combination with immune checkpoint inhibitors (ICPI). (**A**) C57BL6/J mice (*n* = 4/group) were immunized s.c. with 2 nanomoles of free OVA, OVA conjugated to CIRP (OVA-CIRP), OVA plus CIRP (2 or 10 nanomoles each). One week later immune responses in the spleen were measured by IFN-gamma ELISPOT after stimulation with different OVA antigens. (**B**) OVA-CIRP was used as immunogen alone or in combination with ICPI anti-CTLA-4, anti-PD-1, or both antibodies. Responses against OVA protein, CD4 T-cell epitope OVA(323–339), dominant CD8 T-cell epitope 257–264, and subdominant CD8 T-cell epitopes 55–62 and 176–183 were measured as in A. *, *p* < 0.05; **, *p* < 0.01; ***, *p* < 0.001.

To increase efficacy of therapeutic vaccination, and based on our data using a monoepitopic CIRP-based vaccine containing only the immunodominant OVA(257–264) peptide from OVA [18], we designed and produced a new vaccine with broader epitopic repertoire encompassing full OVA fused to CIRP (OVA-CIRP) (Figure S1A). Conjugation of OVA to CIRP clearly promoted anti-OVA immunity, since vaccination with OVA-CIRP induced not only CD8 responses against immunodominant OVA(257–264) CD8 T-cell epitope, but also CD4 responses against OVA(323–339) epitope as well as against full OVA (Figure 1A). By contrast, free untargeted OVA barely induced responses. Co-administration of OVA with a free equimolar amount of CIRP (2 nanomoles) or even a higher CIRP amount (10 nanomoles) was not able to induce any response above those induced by free OVA, indicating that conjugation of OVA to CIRP was necessary.

Besides rescuing already existing exhausted responses, ICPI may also help by enhancing naive T-cell priming. We thus tested the effect of the already approved combination of anti-anti and anti-CTLA-4 inhibitors during immunization with OVA-CIRP. Although single PD-1 blockade provided some beneficial effect, the best results were obtained by combined blockade of PD-1 and CTLA-4, improving the activation of responses not only against immunodominant peptides OVA(257–264) and OVA(323–339), but also against subdominant CD8 epitopes OVA(55–62) and OVA(176–183) (Figure 1B), suggesting that this combination would have a stronger antitumor effect.

### 2.2. Therapeutic Vaccination with a CIRP-Containing Immunogen Increases the Efficacy of ICPI

**Figure 2:**
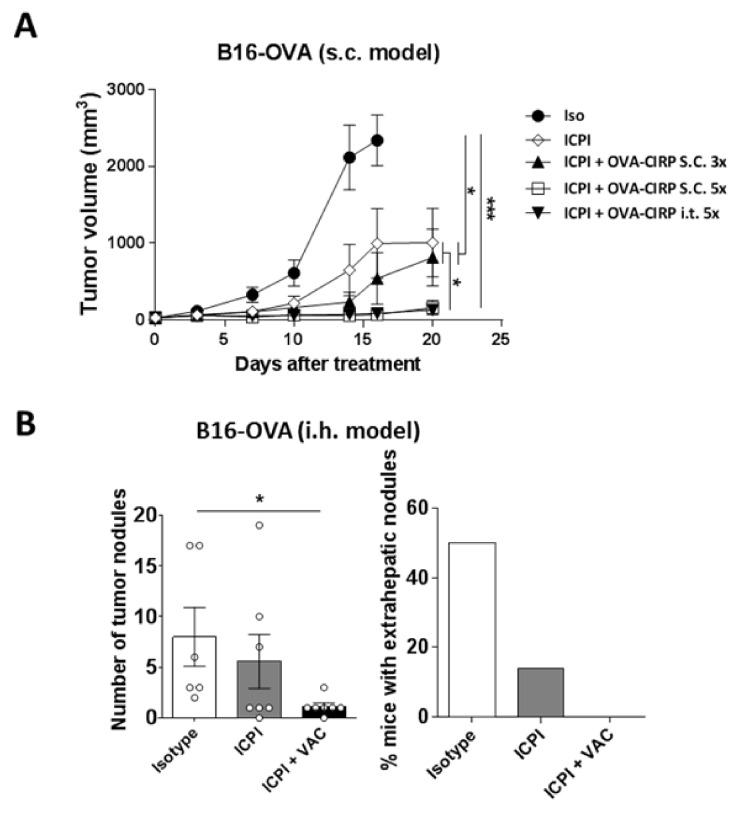
Immunization with OVA-CIRP enhances therapeutic responses induced by ICPI in subcutaneous and intrahepatic tumors. (**A**) C57BL6/J mice (*n* = 6/group) bearing 5 mm subcutaneous B16-OVA tumors were treated with antibodies at days 0, 7, and 14 (Isotype, Iso; anti-CTLA-4 + anti-PD-1, ICPI) with or without OVA-CIRP vaccine administered subcutaneously or intratumor, 3 or 5 times. Tumor volume was measured twice/week. (**B**) B16-OVA cells were injected in the liver of C57BL6/J mice and four days later they received control (*n* = 6) or ICPI antibodies (*n* = 7), or ICPI plus OVA-CIRP vaccine administered s.c. 5 times (*n* = 7). Three weeks later livers were examined, analyzing the number of tumor hepatic nodules as well as the percentage of mice without extrahepatic tumor nodules. *, *p* < 0.05; ***, *p* < 0.001.

Local intratumor vaccination has shown superior therapeutic effect when compared with distal subcutaneous immunization [20]. Despite the common use of intrahepatic percutaneous therapies in HCC [21], intratumor vaccination carries some risks and consumes more health resources than standard vaccination. Therefore, before using the therapeutic combination of vaccine and ICPI in a liver tumor model, we assessed in the subcutaneous B16-OVA tumor model whether distal vaccine administration had equivalent effect to intratumoral vaccination. ICPI administration induced a delay in tumor growth as compared with control animals. However, its combination with OVA-CIRP vaccine strongly repressed tumor growth, mainly when increasing the vaccination schedule from 3 to 5 administrations (Figure 2A). Interestingly, administration of this vaccination schedule at a distal subcutaneous site behaved similarly to intratumoral administration, suggesting that this vaccination protocol could potentially be applied to non-accessible tumors such as those found in the liver.

With these results, we moved to an intrahepatic tumor model established by injecting B16-OVA tumor cells in the liver. After tumor inoculation, mice were randomized in groups receiving isotype control antibodies, ICPI or ICPI combined with the vaccine, according to the same protocol used in the subcutaneous tumor. At day 25 mice were sacrificed and tumors analyzed. Although most mice still had tumors, the number of tumor nodules in mice treated with the combination (range 0–3), but not in those treated with IPCI (range 0–19), was significantly lower (*p* = 0.02) than in control mice (range 2–17) (Figure 2B). Interestingly, mice treated with the combination did not have extrahepatic tumor nodules, whereas 50% and 14% of control mice and mice treated with ICPI, respectively, had these extrahepatic tumors. These results suggest that addition of the subcutaneous vaccine to ICPI enhanced their antitumor effects, even in intrahepatic tumors.

### 2.3. Vaccination Induces Peripheral Antitumor Immunity but Weakly Rescues the Exhausted Phenotype Displayed by Hepatic Tumor-Infiltrating Lymphocytes

**Figure 3:**
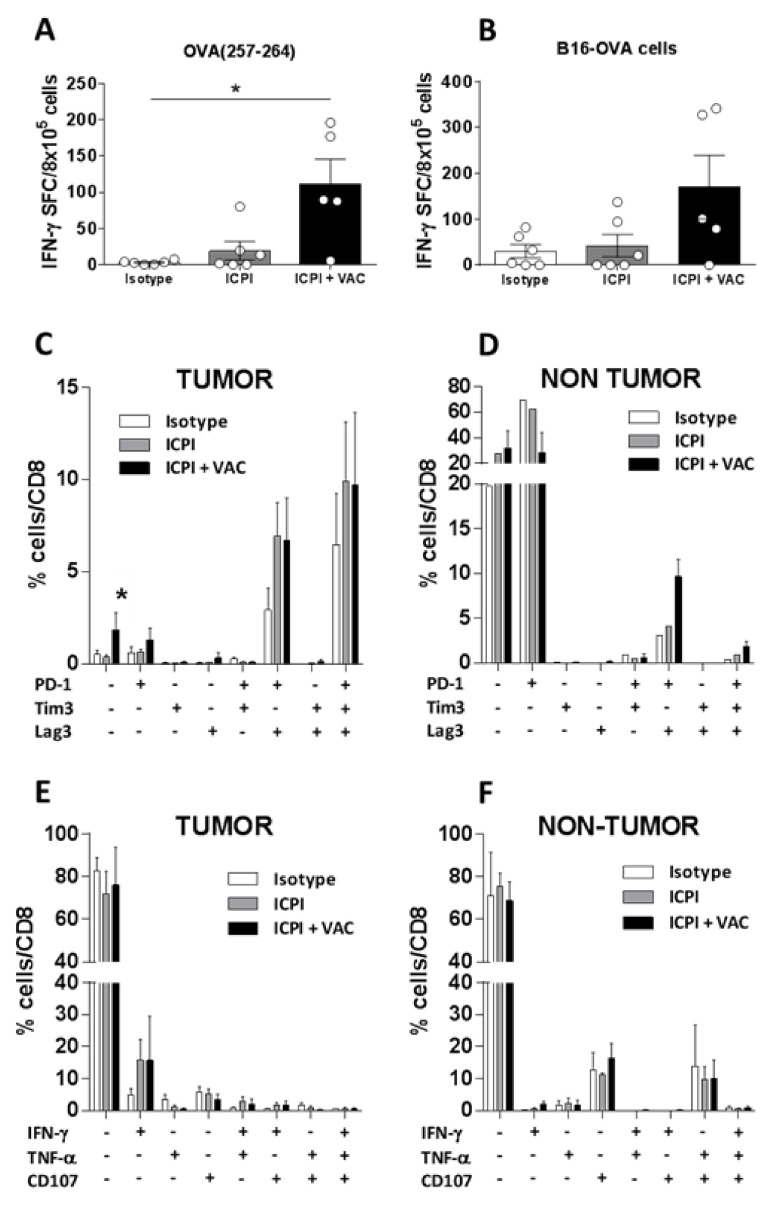
Combined treatment with OVA-CIRP and ICPI promotes antitumor T-cell responses which are exhausted in the tumor. Spleens from mice (*n* = 5–6) with hepatic B16-OVA tumors treated with isotype control antibodies, ICPI or ICPI plus vaccine were obtained at day 25 after treatment, homogenized and cells stimulated with peptide OVA (257–264) (**A**) or with irradiated tumor cells (**B**). In both cases, responses were measured by ELISPOT. Livers from 3 representative animals from each group were also obtained and CD8 T-cells specific for OVA (257–264) in tumor (**C**) and non-tumor liver tissue (**D**), labelled as Tet^+^, were analyzed by flow cytometry, determining the combined expression of inhibitory receptors PD-1, Tim-3 and Lag3. Intrahepatic lymphocytes from tumor (**E**) and non-tumor (**F**) tissue were stimulated with PMA and ionomycine and combined effector functions (expression of cytokines IFN-γ and TNF-α, and of the cytotoxicity marker CD107) were determined by flow cytometry 4 h later. *, *p* < 0.05.

To understand the therapeutic effect obtained in the hepatic tumor model, we analyzed different immune-related parameters. ELISPOT assays measuring splenic IFN-γ-producing cells demonstrated that animals receiving the combination (*p* = 0.02), but not those treated with ICPI alone, had more CD8 T-cells recognizing OVA (258–264) epitope than control mice (Figure 3A). Similarly, the number of lymphocytes recognizing tumor cells was also higher in the combination group (Figure 3B), but it did not reach statistical significance (*p* = 0.058; ICPI + VAC vs. Isotype). In addition to splenic lymphocytes, tumor-infiltrating lymphocytes were also studied. Most tumors contained between 15–25% of infiltrating CD45^+^ leukocytes, with no statistically significant differences in the proportions of CD4, CD8, B cells, or NK cells between groups (Figure S2A–E). Moreover, by using MHC tetramers loaded with OVA (257–264) peptide (Tet), we again observed similar proportions of these tumor antigen-specific lymphocytes (Figure S2F). Analyses of the expression of inhibitory receptors PD-1, Tim3, and LAG-3 in 3 representative mice from each group showed that most of these OVA(258–264) Tet^+^ tumor-infiltrating cells expressed all three receptors, followed by cells expressing PD-1 and LAG-3, and a minority of cells expressing only PD-1 or none of these receptors (Figure 3C). Although for most cell subsets, no clear differences in these parameters were observed between treatment groups, the combination group had a higher proportion of triple-negative cells than control group (*p* = 0.026) and ICPI group (*p* = 0.017). Analyses of tumor-infiltrating OVA (258–264) Tet^−^ cells showed that the majority of these cells were negative for inhibitory receptors (Supplementary Figure S3). There were also some, but fewer, triple-positive and PD-1^+^LAG-3^+^ cells in the OVA (258–264) Tet^−^ subset, presumably those cells specific for other tumor antigens. Interestingly, when considering non-tumor hepatic tissue, OVA (258–264) Tet+ cells revealed an opposite image to tumor tissue, with most cells being triple-negative or only expressing PD-1 (Figure 3D), suggesting a lower degree of activation/exhaustion in the absence of antigen.

Once identified the inhibitory receptor profile of tumor-infiltrating T-cells we studied their functional properties, by stimulating them with PMA/Ionomycin. About 70–80% of tumor-infiltrating CD8 T-cells did not express IFN-γ, TNFα or the degranulation marker CD107, with only a small subset (about 15%) producing only IFN-γ in ICPI and combination groups (Figure 3E). The same triple-negative subset predominated in non-tumor tissue (Figure 3F). All these results suggest that, although combined treatment enhances tumor antigen-specific lymphocytes in the periphery, there is a weaker effect in the intrahepatic tumor compartment. Most of these cells have an exhausted phenotype, characterized by the expression of several inhibitory receptors and the lack of capacity to produce antitumor cytokines. In this context, the combined treatment only affects at the level of enhancing the less exhausted triple-negative subset. 

### 2.4. The CIRP-Based Vaccine Enhances Therapeutic Responses of ICPI-Based Protocols in an HCC Model 

With the results obtained in the intrahepatic B16-OVA model, we next moved to an additional orthotopic HCC model, induced after injection of PM299L cells [22]. These cells were obtained from tumors generated after hydrodynamic injection of plasmids encoding MYC and an activated beta-catenin version, in addition to luciferase and model antigens SIYRYYGL (SIY), OVA (257–264), and OVA(323–339) [23]. Original autochthonous liver tumors were characterized by reduced DC infiltration and concomitant impaired T-cell activity, showing resistance to anti-PD-1 therapy, constituting thus a suitable model for immune enhancement with vaccination protocols. 

**Figure 4:**
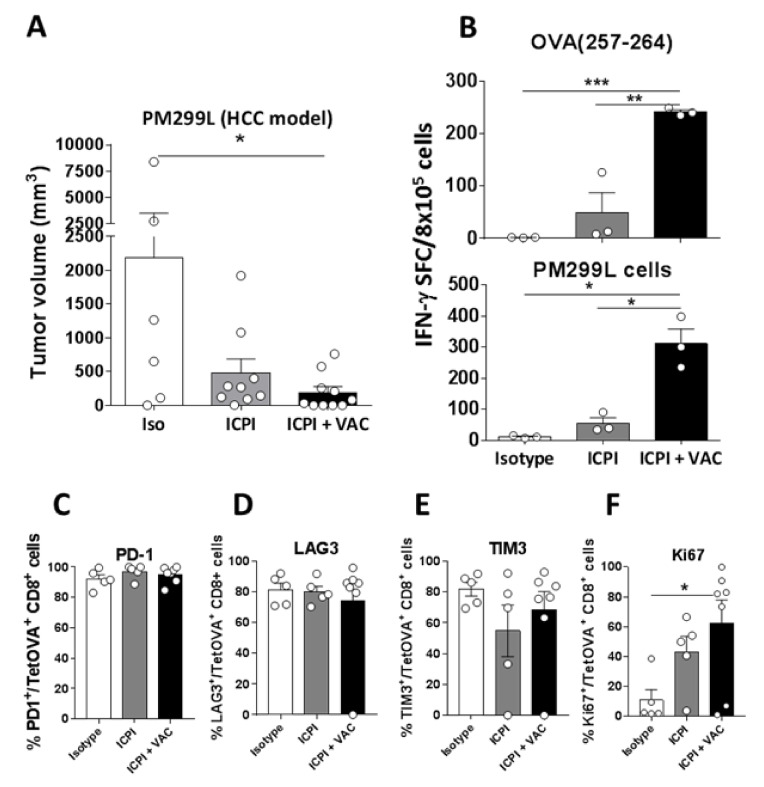
The CIRP-based vaccine enhances therapeutic and immune responses of ICPI-based protocols in mice with HCC. (**A**) C57BL6/J mice (*n* = 6–10/group) were injected in the liver with 5 × 10^4^ PM299L HCC cells. Four days later they received control or ICPI antibodies, or ICPI plus OVA-CIRP vaccine administered s.c. 5 times. Three weeks later livers were examined, analyzing the tumor volume. (**B**) Splenocytes from mice shown in A were stimulated with peptide OVA (257–264) or PM299L tumor cells and immune responses were analyzed by ELISPOT. (**C**–**E**) Expression of ICP PD-1, LAG3, and TIM3 was measured in tumor-infiltrating TetOVA^+^ CD8 T-cells in treated mice. (**F**) Proliferation (Ki67 expression) of TetOVA+ CD8 T-cells. *, *p* < 0.05; **, *p* < 0.01; ***, *p* < 0.001.

Four days after intrahepatic cell injection, luciferase analysis demonstrated that all mice had detectable tumors, they were randomized and treatment started. At day 25 they were sacrificed and tumors analyzed. Mice with tumor presented single masses, therefore we measured tumor volume as a readout of treatment efficacy. We found an important reduction in both ICPI and combination groups, reaching statistical significance only in the latter (Figure 4A). Interestingly, 5 out of 10 treated mice in the combination group had no observable tumors, whereas all mice in the ICPI group still had small but detectable tumors. 

Analyses of antitumor immunity, as occurred in the B16-OVA model, revealed the presence of strong CD8 responses in the spleen recognizing OVA (257–264) or tumor cells (Figure 4B). In the case of tumor-infiltrating leukocytes, we did not observe clear differences between treatment groups when analyzing the different T-cell subsets (data not shown). Regarding T-cells, as in the previous hepatic model, most of tumor antigen-specific T-cells (OVA (258–264) Tet^+^ cells) displayed a prominent expression of inhibitory receptors, suggesting an exhausted phenotype (Figure 4C–E). However, vaccination induced a higher proportion of Ki67^+^ proliferating OVA (258–264) Tet^+^ T-cells (Figure 4F). These results confirm in a new model that vaccination induces more potent antitumor responses, associated with increased ICPI therapeutic efficacy. Nevertheless, despite this stimulatory capacity, many tumor-infiltrating T-cells remain positive for checkpoint molecules.

### 2.5. Immunization with Human HCC Antigen Glypican 3 Conjugated to CIRP (GPC3-CIRP) Induces Anti GPC3 Immunity, Enhanced by Combination with ICPI

**Figure 5:**
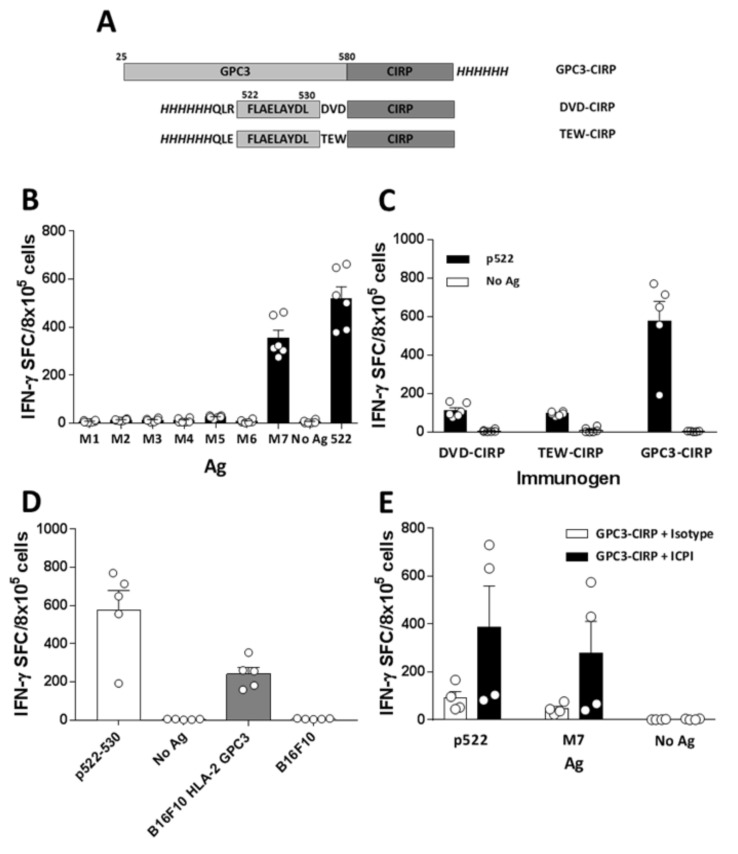
Immunization with human HCC antigen GPC3 conjugated to CIRP (GPC3-CIRP) induces anti GPC3 immunity. (**A**) Scheme of the different CIRP-based vaccines containing GPC3-associated antigens. Antigens correspond to light grey boxes; DVD-CIRP and TEW-CIRP immunogens also display additional flanking amino acids. In all immunogens, 6xHis tags are shown italicized. (**B**) HHD-DR1 mice (*n* = 6) were immunized with 2 nanomoles of HCC Ag GPC3 conjugated to CIRP (GPC3-CIRP) and one week later splenocytes were stimulated with 7 peptide pools encompassing the whole GPC3 sequence (M1 to M7) as well as with peptide GPC3(522–530) (contained in M7) and T-cell responses were evaluated by ELISPOT. (**C**) Immunogenicity of entire GPC3-CIRP (*n* = 5) was compared with shorter Ag versions containing p522–530 plus different flanking regions conjugated to CIRP (DVD-CIRP and TEW-CIRP) (*n* = 6 in both groups). Responses were evaluated in ELISPOT assays using peptide 522–530. (**D**) Recognition of tumor antigens by lymphocytes from HHD-DR1 mice (*n* = 5) immunized with GPC3-CIPR was analyzed in ELISPOT assays by using parental B16F10 cells and B16F10 tumor cells transduced with human HLA-A2 molecules and with GPC3 Ag. (**E**) GPC3-CIRP vaccine was administered with control or IPCI antibodies (*n* = 4 in both groups) and responses against p522–530 and M7 pool were evaluated by ELISPOT.

To translate these results to the human HCC setting, we focused on CIRP-based vaccines containing the human HCC antigen glypican 3 (GPC3) [24]. As a first approach, we designed an immunogen containing the full GPC3 sequence linked to GPC3 (GPC3-CIRP) (Figure 5A). Immunization of HHD-DR1 mice (transgenic for human MHC class I and class II molecules HLA-A2*01 and HLA-DRB1*01, respectively) with GPC3-CIRP induced potent T-cell responses, that were mapped using a panel of 7 pools with overlapping 20-mer peptides. This response was mainly directed against pool M7, that contains the already described HLA-A2*01-restricted epitope 522–530 [25] (Figure 5B). Due to the immunodominance of this epitope, and since we had demonstrated good antitumor activity of monoepitopic CIRP-based vaccines containing a single CD8 epitope [18], we also designed and produced two new immunogens containing only the minimal 522–530 epitope. In addition to this sequence and in order to optimize antigen processing, we added two flanking regions of three amino acids: (i) those present in the natural GPC3 sequence surrounding peptide 522–530 (DVD-CIRP vaccine) or (ii) those amino acids flanking the OVA(257–264) epitope (TEW-CIRP vaccine) (Figure 5A; Supplementary Figure S1B). These vaccines also induced responses that recognize peptide 522-530; however, they were not as potent as those induced by GPC3-CIRP (Figure 5C). Responses elicited by GPC3-CIRP vaccine not only recognized synthetic 522–530 peptide but could also detect the naturally processed epitope presented by tumor cells, since they were activated by B16F10 tumor cells expressing HLA-A2*01 and GPC3, but not by parental B16F10 cells (Figure 5D).

Finally, as in the previous OVA-CIRP model, we tested the combined effect of the vaccine plus ICPI in terms of immunogenicity. As shown in Figure 5E, combination of GPC3-CIRP with anti-PD-1 and anti-CTLA-4 antibodies induced stronger responses against M7 pool and peptide 522–530 than GPC3-CIRP alone, confirming the suitability of this combination for future immunotherapies. 

### 2.6. Combination of Vaccine with ICPI Does not Enhance Liver Toxicity in Mice

**Figure 6:**
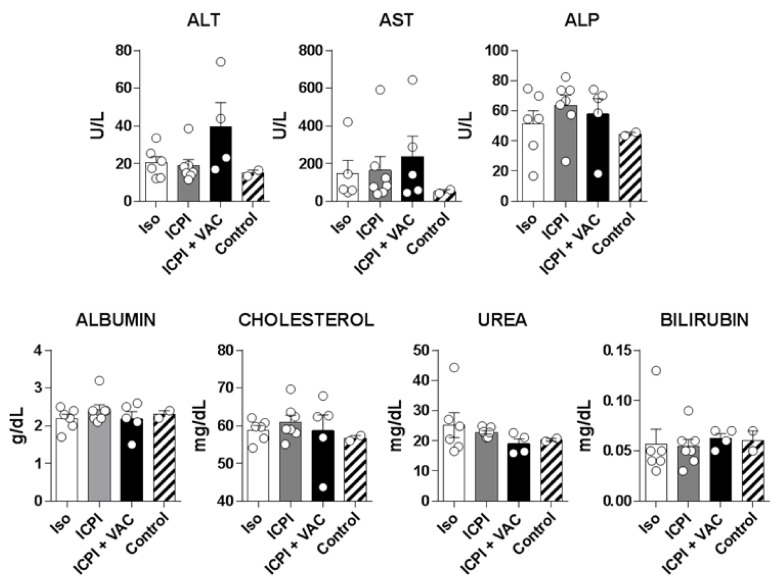
Combination of the CIRP-based vaccine with ICPI does not enhance liver toxicity. C57Bl6/J mice (*n* = 5/group) with hepatic B16-OVA tumors were treated with isotype or ICPI antibodies, with the combination of ICPI and OVA-CIRP and serum was obtained after treatment completion. As control, serum was obtained from two mice without tumors. Hepatic enzymes and liver-associated markers were measured in sera.

ICPI-based therapies have been associated with the presence of different immune-related adverse events (irAE), including those affecting the liver in HCC patients, which may be aggravated when combined with other ICPI or additional immunostimulatory molecules [26]. CIRP has been described as an inflammatory molecule produced under stress conditions that, when present at higher serum concentrations, may promote inflammatory damage [19]. Hence, we studied the potential hepatotoxicity of the combination in mice with hepatic tumors. Serum was obtained from mice with B16-OVA tumors once they completed treatment, as described for Figure 2B, as well as from control mice without tumors, and the levels of liver enzymes and other hepatic markers were analyzed. No statistically significant differences were observed between groups (Figure 6). Except for a mouse in the combination group that had higher ALT levels, remaining mice had similar levels to the other groups, discarding a superior toxicity induced by the combination with the CIRP vaccine, at least in mice. 

## 3. Discussion

The limited response rates obtained in HCC by using immunotherapeutic protocols based on ICPI suggest that new combinations are necessary to benefit a higher proportion of patients [27,28,29]. Since poorer responses to ICPI are associated with a lower presence of tumor-infiltrating lymphocytes specific for tumor antigens [12], many combinatorial strategies are aimed at enhancing the number of these cells. Currently approved antitumor therapies, such as chemo- and radiotherapy, have shown immune-mediated effects, that may partially explain their therapeutic efficacy, leading thus to their use as combinatorial partners with ICPI [30,31]. Likewise, in the case of HCC, combinatorial protocols (e.g., TACE plus anti-CTLA-4) [32] have been reported and an important number of combinations are under investigation [33]. However, in addition to these combinations, vaccination emerges as a highly specific strategy to enhance antitumor immunity against relevant antigens of choice [34]. With this purpose, we have tested a combinatorial protocol based on the administration of a CIRP-based vaccine plus ICPI anti-CTLA-4 and anti-PD-1. We previously demonstrated that conjugation of a monoepitopic CD8 peptide antigen to CIRP (an endogenous TLR4 ligand expressed under stress conditions), confers immunogenicity to these antigens [18]. Here we demonstrate that CIRP can harbor larger antigens, inducing polyepitopic CD4 and CD8 responses, suggesting that this vaccine could be a suitable candidate for combination with ICPI.

Combination of vaccines and ICPI provides a two-sided benefit in tumor therapy: first, vaccination promotes the expansion of the pool of effector antigen-specific cells that will be amenable to rescuing by ICPI in the tumor, and second, ICPI increase vaccine immunogenicity, by reducing immunosuppressive mechanisms [16,17] operating during naive T-cell priming. In this second scenario, we have seen that both in naive tumor-free and in tumor-bearing mice, combination of vaccines (either OVA-CIRP or GPC3-CIRP) are more immunogenic in the presence of anti-CTLA-4 and anti-PD-1 antibodies. Vaccination upregulates expression of molecules targeted by these antibodies on T-cells, as well as of PD-L1 on antigen-presenting cells, and as we have demonstrated in other vaccination settings, blockade of these and other immunoregulatory factors [18,35] results in increased T-cell responses.

As previously mentioned, combinatorial therapies containing ICPI include elements aimed at increasing tumor inflammation. For this purpose, many of them rely on procedures directly affecting the tumor, such as radiotherapy or locally administered compounds. Similarly, we had previously shown that CIRP-based vaccines had a good efficacy when administered intratumorally into subcutaneous tumors [18]. In addition to generation of T-cells with antitumor activity, vaccine administration at the tumor site may activate innate immunity locally, leading to enhanced responses mediated by macrophages or NK cells, as described for other TLR4 ligands [36]. However, for future use in an internal tumor such as HCC, we tested whether distally administered CIRP vaccines would retain their efficacy. By using a subcutaneous model, we demonstrated that, when combined with ICPI, a distant and subcutaneous vaccination had similar therapeutic efficacy, suggesting that it could be useful for HCC and leading thus to the next series of experiments on hepatic tumors.

Therapeutic experiments in tumors in the liver showed that, according to our hypothesis of immune enhancement induced by the vaccine, the combination group compared better to control animals than ICPI alone in the B16-OVA and PM299L models. Indeed, in both cases, we saw clear induction of tumor-specific immunity in the combination group, mainly when analyzing peripheral responses in the spleen. However, analysis of immune parameters in the tumor revealed a different scenario, where the effect of the vaccine was not as clear as that observed in the spleen. The study of immune checkpoint receptors and effector functions in tumor-infiltrating lymphocytes showed that most cells had an exhausted phenotype, with a poor capacity to produce effector cytokines. Indeed, vaccination barely changed this, with the exception of a higher number of CD8 T-cells negative for inhibitory checkpoint receptors, and a higher proportion of tumor-antigen specific T-cells with proliferative capacity, both indicative of non-exhausted cells. Interestingly, combination of anti-CTLA-4 and antiPD-L1 in HCC patients resulted in an increase of proliferating CD8 T-cells [37], suggesting that addition of a vaccine may promote even a higher proliferative burst, as observed in our results. These results suggest that vaccination may generate new tumor-specific lymphocytes with effector functions, but the immunosuppressive tumor microenvironment either does not allow their infiltration into the tumor or alternatively, promotes their exhaustion. Besides PD-1, targeted in our ICPI therapy, we have observed generalized Tim3 and Lag3 expression on tumor-specific lymphocytes, as already reported in HCC patients [38,39]. Therefore, ICPI antibodies used in this work may not be sufficient to release T-cells from tumor-imposed brakes, suggesting that blockade of additional targets may improve therapeutic efficacy. Indeed, ex vivo experiments have shown that combined, rather than single blockade, of PD-1, Lag3 and Tim3, more efficiently rescues cytokine production and proliferative capacity of exhausted tumor-infiltrating CD4 and CD8 T-cells [38]. More importantly, in the clinical HCC setting, efficacy of initial ICPI therapies (based on single blockade of CTLA-4 or PD-1), have increased response rates from 15–20% to 30–35% when using anti-PD-1/PD-L1 combined with anti-CTLA-4 or anti-VEGF [9,10]. These results are encouraging because they confirm that targeting of a higher number of molecules promotes better responses. However, at the same time, they show that an important proportion of patients still remain as non-responders, suggesting that vaccination, in the presence of simultaneous blockade of these molecules, may increase their efficacy. In this regard, in addition to typically immunomodulatory molecules such as PD-1/PD-L1 and CTLA-4, VEGF has emerged as an interesting target. Besides its role in angiogenesis, VEGF has important immunomodulatory properties, such as its inhibitory role in dendritic cells and the recruitment of immunosuppressive subsets, among others [40], transforming thus the tumor microenvironment. Indeed, it has been recently reported in a preclinical HCC model [41] that dual PD-1 and VEGFR blockade increases CD8 T-cell infiltration, associated with a higher therapeutic efficacy, suggesting that vaccine-induced T-cells may benefit from this property of VEGF blockade.

Having demonstrated the efficacy of the CIRP vaccination platform, we translated this to a human setting, designing different immunogens based on the human HCC antigen GPC3, which is expressed in 80% of HCC tumors [24]. Testing of a vaccine containing the full GPC3 sequence in humanized mice expressing HLA-A2*01 and HLA-DRB1*01 demonstrated strong responses against the immunodominant HLA-A2*01-restricted epitope 522–530 [25]. Interestingly, this response was clearly better than those induced by two other monoepitopic vaccines containing only 522–530 peptide. Although we did not detect measurable CD4 responses when screening the whole GPC3 sequence, we hypothesize that such a large antigen may harbor CD4 T-cell epitopes that help inducing CD8 T-cell responses against 522–530 epitope. Indeed, in the case of the model antigen OVA we have seen that CIRP based vaccines can induce both CD8 and CD4 responses. These results suggest that conjugation of CIRP to large tumor antigens should be preferred, to enhance the possibility of including CD4 epitopes that provide T-cell help to other immune subsets, and to include several CD8 and CD4 epitopes presentable by different HLA alleles, broadening thus the proportion of individuals in a heterogeneous population.

Finally, irAE have been reported in ICPI-treated cancer patients, including those with HCC, that may involve several organs [26]. Liver toxicity, not uncommon in these irAE, is aggravated in the case of HCC, since tumors usually develop on a cirrhotic liver [26]. The presence of irAE increases when using combined therapies that enhance immunostimulation [42]. Therefore, we checked whether in our protocol, combination of ICPI with CIRP, implicated in inflammation-associated events such as hemorrhagic shock and sepsis [19], would induce hepatic adverse events. However, we did not observe any effect of our CIRP-based vaccine on hepatic toxicity after a full treatment protocol in mice with hepatic tumors, suggesting that its use could be safe in HCC patients. Although the absence of a damaged cirrhotic liver in our tumor models might be partially responsible for the lack of toxicity, we favor the hypothesis that the administration protocol, where the vaccine is injected s.c. at a local site instead of systemically or in the damaged liver, avoids the generation of irAE. Indeed, those models where CIRP administration results in inflammatory conditions [19] involve systemic administration that clearly enhances CIRP serum levels, which are not presumably reached with our local vaccination protocol.

Taken together, our results reveal that CIRP-based vaccines are suitable tools to enhance therapeutic efficacy of ICPI in HCC. This is associated with the generation of stronger antitumor T-cell immunity. However, despite this priming capacity of the vaccine, weak effects are seen in tumor-infiltrating lymphocytes, which display an exhausted phenotype, suggesting that future combinatorial strategies should consider blockade of additional targets. 

## 4. Materials and Methods 

### 4.1. Immunogens

Plasmids encoding OVA-CIRP (containing the full OVA sequence), DVD-CIRP (containing the 522–530 GPC3 epitope plus three natural flanking amino acids at each extreme) and TEW-CIRP (containing the 522–530 GPC3 epitope plus three flanking amino acids surrounding the OVA(257–264) epitope at each extreme) were obtained from Genscript. To generate these plasmids, antigens were cloned into the previously described pET14b plasmid that encodes OVA(257–264) peptide fused to CIRP and a 6 Histidine tail [18] by exchanging this peptide with the new antigens. BL21(DE3) bacteria were transduced with the plasmids and induction of protein expression with IPTG and purification was carried out as described [18]. CIRP protein without any antigen has been previously described [18]. Endotoxin was removed by extensive washing during the purification steps, resulting in levels below 10 ng endotoxin/mg of protein, as tested by Quantitative Chromogenic Limulus Amebocyte Lysate assay (Lonza, Barcelona, Spain). 

GPC3-CIRP protein was obtained from Genscript. It contained the full GPC3 sequence (except the 24 amino acids corresponding to the signal peptide, as well as two modifications to alanine at residues 358/359 at the furin cleavage site) plus CIRP and a C-terminal 6 histidine tail. This sequence was cloned in the pFastBac1 plasmid for expression in insect cells.

Recombinant endotoxin-free OVA protein (Endograde) was purchased from Hyglos (Bernried, Munich area, Germany). OVA peptides 257–264, 323–339, 55–62 and 176–183, and GPC3 522–530 peptide, were from Genecust (Luxemburg, Boynes France). A panel of 20-mer peptides with a 10 amino acid overlap spanning the entire GPC3 sequence was synthesized by using an automated Appex396 peptide synthesizer (Aapptech, Louisville, KY, USA). 

### 4.2. Mice

C57BL/6J mice (eight-weeks old) were obtained from Envigo. HHD-DR1 mice (B2m^tm1Unc^ H2-Ab1^tm1Doi^ Tg(HLA-A/H2-D/B2M)1Bpe Tg(HLA-DR1)/Orl), encoding human HLA-A2*01 and HLA-DRB1*01 were obtained from Dr. F. Lemmonier (Paris, France) and breed. They were maintained in pathogen free conditions according to the guidelines for institutional animal care and use committee (protocol 128-14). 

### 4.3. Tumor Cell Lines

B16-OVA and B16.F10 melanoma cells (obtained from Dr. G. Kroemer; Paris, France) and B16.HLA-A2 (obtained from Dr. R. Alemany; Barcelona, Spain), were grown in DMEM containing 10% FBS and antibiotics. B16.HLA-A2 expressing GPC-3 were generated by retroviral (RV) infection with a RV expressing hGPC-3. Briefly, hGPC-3 was cloned in plasmid MSCV IRES Thy1.1. (kindly provided by Dr. Anjana Rao, la Jolla Institute, CA) to generate MSCV hGPC3-IRES-Thy1.1 plasmid. B16.HLA-A2 were infected with the RV and transduced cells (Thy1.1^+^) were sorted by flow cytometry (BD FACSAria cell sorter). PM299L HCC cells were obtained after injection of C57BL/6 mice with plasmids *pT3-EF1a-MYC-IRES-luciferase-OS* (*MYC-lucOS*), *pT3-N90-CTNNB1 (CTNNB1) and SB13* transposase–encoding plasmid as described [23]. Growing liver tumors were excised, homogenized, cultured in vitro and further passaged in vivo in C57BL/6 mice to yield PM299L cells.

### 4.4. Vaccination to Evaluate Protein Immunogenicity

Mice were immunized by s.c. administration of 2 nanomoles of recombinant proteins diluted in PBS. In some cases, when co-administering unbound CIRP, immunization included 2 or 10 nanomoles. In experiments testing the effect of ICPI, the day of immunization mice also received an i.p. injection with 100 μg of isotype control antibodies (clone 2A3), anti-CTLA-4 (clone 9D9), anti-PD-1 (clone RMP1-14) or a combination of ICPI (all from BioXcell, NH, USA). One week after immunization, mice were sacrificed and spleens homogenized to obtain cell suspensions. 

### 4.5. ELISPOT

Responses induced by vaccination were measured by using an IFN-γ ELISPOT Set (BD-Biosciences, Franklin Lakes, NJ, USA). Briefly, splenocytes (8 × 10^5^ cells/well) were cultured in ELISPOT plates and stimulated with OVA or GPC3 CD4/CD8 epitope peptides (10 μg/mL), OVA protein (10 μg/mL), GPC3 peptide pools (1 μg/mL of each peptide) or irradiated tumor cells (8 × 10^4^ cells/well). One day later plates were washed, developed and spot-forming cells were counted automatically.

### 4.6. Tumor Treatment Experiments 

For the s.c. B16-OVA model, mice were injected with 5 × 10^5^ cells and when tumor reached 5 mm in diameter (one week later, approximately, day 0) they were randomized and treated with vaccination (OVA-CIRP, 2 nanomoles at days 0, 4, 7, 11 and 14 (5×) or at days 0, 7 and 14 (3×); by s.c. or intratumor routes), antibodies against PD-1, CTLA-4 (ICPI) or isotype control (100 μg/mouse) at days 0, 7 and 14, by i.p. route, or the combination. Tumor volume was calculated using the formula: V = (length × width2)/2. Mice were euthanized after three weeks of treatment or if tumors reached 17 mm in diameter.

For the intrahepatic models, 5 × 10^5^ B16-OVA cells or 5 × 10^4^ PM299L cells were injected after laparotomy. Treatments started at day 4. In the case of PM299L tumors, due to the lower number of cells administered, bioluminescence was measured at this time point, to check tumor growth and randomize animals into treatment groups. Mice received isotype or ICPI with or without s.c. vaccination using 5 vaccine administrations given as in the s.c. B16-OVA model. At day 25 mice were sacrificed, tumor nodules were counted, and volume was measured. 

### 4.7. Flow Cytometry

Tumors were incubated with collagenase/DNase for 15 min, homogenized and first incubated with Fc Block™ (BD-Biosciences) for 10 min. Then, tumor samples were stained with the following antibodies to simultaneously quantify lymphocyte subsets, OVA(257-264) specific CD8 T-cells and expression of immune checkpoint molecules: CD45-APC-Cy7, K^b^/OVA(257-264)-APC, CD4-Alexa 700, CD8-PE, CD3-BUV496, PD1-PerCP/Cy5.5, LAG3-BV650 and TIM3-BV785 (all from Biolegend, San Diego, CA, USA, except CD3-BUV496 which is from BD-Biosciences).

To analyze effector functions cells were stimulated for 4 h with PMA (100 ng/mL)/ionomycin (1 μg/mL) and then stained with antibodies against CD45-APC-Cy7, CD4-Alexa 700, CD8-BV421 CD107a-FITC (all from Biolegend), CD3-BUV496 (from BD-Biosciences). Then cells were fixed and permeabilized and finally stained intracellularly with anti-IFN-γ-PE and TNF-α-BV510.

In all cases the promofluor 840 (maleimide, Promokine, Burlingame, CA, USA) was added to stain dead cells. Samples were acquired with FACSCantoII (Becton Dickinson, Franklin Lakes, NJ, USA) or with Cytoflex (Beckman Coulter, Irving, TX, USA) flow cytometers and analyzed using FlowJo software (Tree Star Inc., Ashland, OR, USA).

### 4.8. Serum Markers

Sera were obtained from tumor-bearing mice after finishing the treatment protocol or from control mice. Levels of liver enzymes Alanine aminotransferase (ALT), Aspartate aminotransferase (AST), Alkaline Phosphatase (ALP), albumin, urea, cholesterol and total bilirubin were determined using a C311 Cobas Analyzer (Roche Diagnostics, Indianapolis, IN, USA) following manufacturer’s instructions.

### 4.9. Statistical Analyses

Data are shown as mean ± SEM. Differences between groups were compared by using Student’s *t*-test and one-way ANOVA with Bonferroni’s multiple comparison test. A *p* value < 0.05 was considered statistically significant. Statistical analysis was performed using the GraphPad Prism software (GraphPad, San Diego, CA, USA).

## 5. Conclusions

Our results show that conjugation of relevant liver tumor antigens to the CIRP platform may be a suitable strategy to generate vaccines that enhance the therapeutic efficacy of ICPI in HCC. This is associated with the generation of stronger antitumor T-cell immunity. However, despite the immunogenicity and antitumor effect of these vaccines, the exhausted phenotype observed in most tumor-infiltrating lymphocytes suggests that blockade of additional targets may be necessary to improve future vaccine-containing combinatorial immunotherapies.

## Supplementary Materials

The following are available online at https://www.mdpi.com/2072-6694/12/11/3397/s1, Figure S1: Expression of CIRP-containing immunogens, Figure S2: Analysis of tumor-infiltrating leukocytes in mice with intrahepatic B16-OVA tumors. Figure S3: Analysis of checkpoint expression in OVA (257–264)Tet- cells in tumors from treated mice.
