# Supplementary material for: Cold-Inducible RNA Binding Protein as a Vaccination Platform to Enhance Immunotherapeutic Responses against Hepatocellular Carcinoma
Source: Cancers (Basel). 2020 Nov 16;12(11):3397. doi: 10.3390/cancers12113397 (PMC7696968; doi:10.3390/cancers12113397)
Supplement: Supplementary file 1 [file cancers-12-03397-s001.pdf]

cancers

Supplementary material

Cold-Inducible RNA Binding Protein as a Vaccination Platform to Enhance Immunotherapeutic Responses Against Hepatocellular Carcinoma

Leyre Silva, Josune Egea, Lorea Villanueva, Marta Ruiz, Diana Llopiz, David Repáraz, Belén Aparicio, Aritz Lasarte-Cia, Juan José Lasarte, Marina Ruiz de Galarreta, Amaia Lujambio, Bruno Sangro and Pablo Sarobe

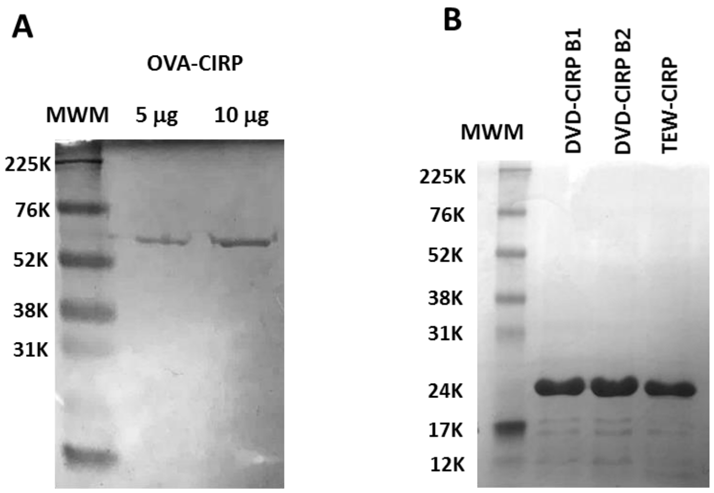

Figure S1. Expression of CIRP-containing immunogens. Protein expression was induced in BL21(DE3) bacteria, and after purification by affinity chromatography, purity was checked. Representative gels with Coomassie staining corresponding to OVA-CIRP (**A**) and DVD-CIRP (batches B1 and B2) and TEW-CIRP (**B**) after protein purification.

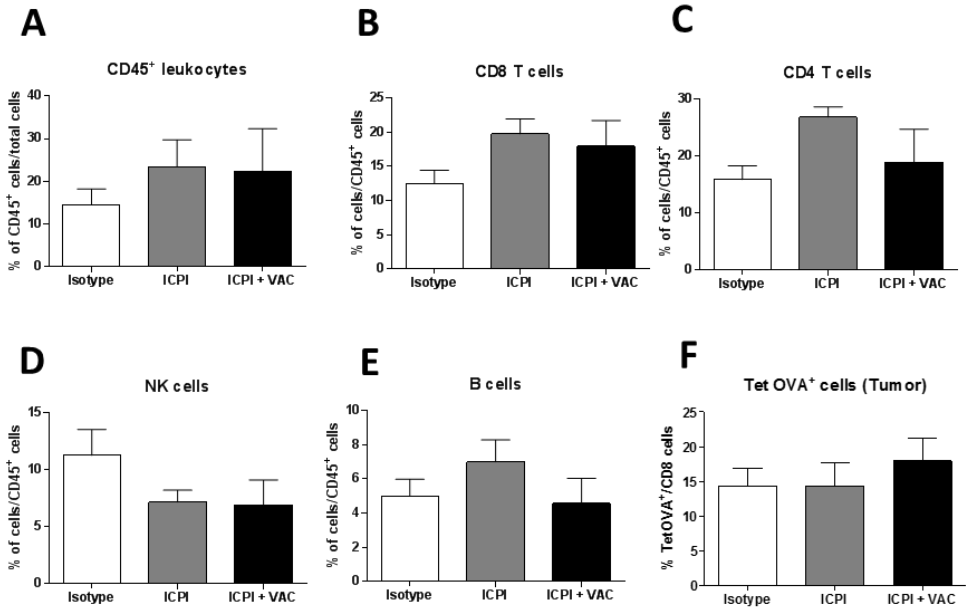

Figure S2. B16-OVA cells were injected in the liver of C57BL6/J mice (n = 6) and one week later they received control or ICPI antibodies, or ICPI plus OVA-CIRP vaccine administered s.c. 5 times. Three weeks later livers tumors were obtained and cells stained with different antibodies to determine the percentage of CD45+ infiltrating leukocytes (**A**), CD8 (**B**) and CD4 (**C**) T cells. NK cells (**D**) and B cells (**E, F**). In the gate of CD8 T lymphocytes, cells were selected based on staining with OVA (257-264) tetramers.

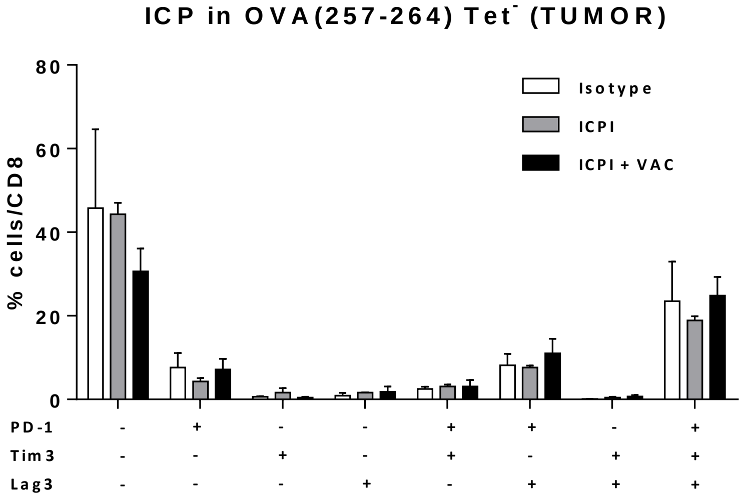

Figure S3. Tumor tissue from mice with hepatic B16-OVA tumors treated with isotype control antibodies, ICPI or ICPI plus vaccine was obtained at day 25 after treatment and homogenized. Cells were stained with antibodies to determine the combined expression of exhaustion markers PD-1, Tim-3 and Lag3 in CD8 lymphocytes non-specific for OVA (257-264), labelled as Tet-.

© 2020 by the authors. Licensee MDPI, Basel, Switzerland. This article is an open access article distributed under the terms and conditions of the Creative Commons Attribution (CC BY) license (http://creativecommons.org/licenses/by/4.0/).
